# A phase 1b randomized, multicenter, dose determination trial of zelpultide alfa (recombinant human surfactant protein D) in preterm neonates at high risk of developing bronchopulmonary dysplasia

**DOI:** 10.3389/fped.2025.1639573

**Published:** 2025-09-12

**Authors:** Almudena Alonso-Ojembarrena, Brenda Poindexter, Samia Aleem, Helen Healy, Marta Aguar-Carrascosa, Elisenda Moliner-Calderón, María del Mar Serrano-Martín, Raquel Arroyo, Maximo Vento

**Affiliations:** ^1^ Neonatal Intensive Care Unit, Puerta del Mar University Hospital, Cadiz, Spain; ^2^ Biomedical Research and Innovation Institute of Cadiz (INiBICA), Puerta del Mar University Hospital, Cadiz, Spain; ^3^ Department of Pediatrics, Emory University and Children’s Healthcare of Atlanta, Atlanta, GA, United States; ^4^ Department of Pediatrics, Duke University Medical Center, Durham, NC, United States; ^5^ Department of Neonatology, Beth Israel Deaconess Medical Center, Boston, MA, United States; ^6^ Neonatal Intensive Care Unit, La Fe University Hospital, La Fe Research Institute, Valencia, Spain; ^7^ Neonatology Unit, Hospital Universitari de la Santa Creu I Sant Pau, Institut de Recerca Sant Pau—IR Sant Pau, Barcelona, Spain; ^8^ Neonatology Intensive Care Unit, Regional University Hospital Málaga, Málaga, Spain; ^9^ Airway Therapeutics Spain SL, Madrid, Spain; ^10^ Instituto de Investigación Sanitaria La Fe (IISLAFE), Valencia, Spain

**Keywords:** SP-D, premature neonates, respiratory distress, bronchopulmonary dysplasia, zelpultide alfa

## Abstract

**Background:**

Bronchopulmonary dysplasia (BPD) ranks among the most severe long-term complications of prematurity. Surfactant protein D, not present in commercial surfactant, regulates the innate immune response of the lungs by clearing infectious pathogens and limiting pulmonary inflammation and inflammatory injury. We aimed to assess the safety and tolerability of zelpultide alfa vs. air-sham when added to the standard of care in preterm neonates at risk of BPD. Efficacy was a secondary outcome.

**Methods:**

A phase 1b, randomized, double-blind, dose-determination study was conducted that enrolled intubated, mechanically ventilated preterm neonates who required ≥1 surfactant treatment within 96 h of birth. Initially, eight subjects [25–28 ^6^/_7_ weeks gestational age (GA)] were randomized 3:1 to receive up to two doses of intratracheal zelpultide alfa at each dosing level (2, 4, or 6 mg/kg) or air-sham 24 h apart. Moreover, 12 additional subjects (23–28 ^6^/_7_ weeks GA) were randomized 3:1 to receive the highest-tolerated dose of zelpultide alfa, or air-sham, once daily for up to 7 days.

**Results:**

In total, 37 subjects were randomized and treated. Zelpultide alfa, at its highest dose of 6 mg/kg, had a favorable safety profile. Furthermore, 92.9% of zelpultide alfa subjects vs. 100.0% of those that received air-sham experienced ≥1 adverse event. The mortality rate was 21% in the zelpultide alfa group and 0% in the air-sham group, although no deaths were related to the study drug. The incidence of BPD was 32.1% vs. 66.7%, the incidence of BPD or death was 54% and 67%, and time on mechanical ventilation was 17.7 vs. 25.8 days in the zelpultide alfa group compared to the air-sham group, respectively.

**Conclusions:**

This study endorses the safety and tolerability of zelpultide alfa up to 6 mg/kg (≤7 days) and reinforces the need for further clinical development of zelpultide alfa as a therapy for preventing BPD.

**Clinical Trial Registration**: https://clinicaltrials.gov/study/NCT04662151?cond=BPD&term=At-100&rank=1, identifier NCT04662151.

## Background

Bronchopulmonary dysplasia (BPD) is a multifactorial chronic respiratory disease that ranks among the most common complications affecting extremely preterm infants and leads to significant comorbidities and mortality (1, 2). BPD is influenced by incomplete or abnormal lung development due to prematurity, injury, infection, and inflammation, which result in inefficient gas exchange and impaired lung mechanics. Furthermore (1, 3), BPD is associated with prolonged hospitalization during the neonatal period, growth failure, and long-term consequences, such as pulmonary and neurodevelopmental impairments (4, 5). The global incidence of BPD has been reported to range from 17% to 75% in extremely preterm infants [<28 weeks gestational age (GA)] (6) and has increased in recent years (7). Clinical management includes the administration of antenatal steroids to the mother, surfactant replacement therapy, postnatal steroid administration (8), caffeine therapy, vitamin A, and gentle mechanical ventilation (1).

Zelpultide alfa (previously AT-100) is a novel, biotechnological therapy with the active ingredient recombinant human surfactant protein D (rhSP-D) and is in development for preventing BPD in preterm neonates. Current surfactant therapies for respiratory distress syndrome do not include surfactant protein D (SP-D), which is involved in regular surfactant lipid structure and lipid recycling (9, 10). SP-D helps regulate the innate immune response of the lungs by clearing infectious pathogens and limiting pulmonary inflammation and inflammatory injury (1). Hence, treating preterm neonates with zelpultide alfa may reduce lung injury and inflammation and lead to a reduction in the incidence or severity of BPD (1, 11). We aimed to assess three dose levels of zelpultide alfa to maximize the benefit-to-risk ratio in extremely preterm neonates who are at high risk of developing BPD. The primary objective was to establish a safety and tolerability profile for the optimal dose of zelpultide alfa. The secondary objective was to evaluate preliminary efficacy outcomes, including the incidence of BPD or death and time on mechanical ventilation.

## Methods

### Trial design

This phase 1b study was conducted at 12 sites in the United States and 10 in Spain. The trial was designed, executed, recorded, and reported in compliance with the principles of the Good Clinical Practice guidelines, and monitored by an appropriate Data Safety Monitoring Committee (DSMC). The DSMC had four voting members, including a neonatologist, a pharmacologist, and a biostatistician, all of whom were independent of the Sponsor and the Sponsor's chief medical officer. The study aimed to evaluate the safety and tolerability of the optimal dose of zelpultide alfa in preterm neonates at high risk of developing BPD compared to air-sham.

**Figure 1:**
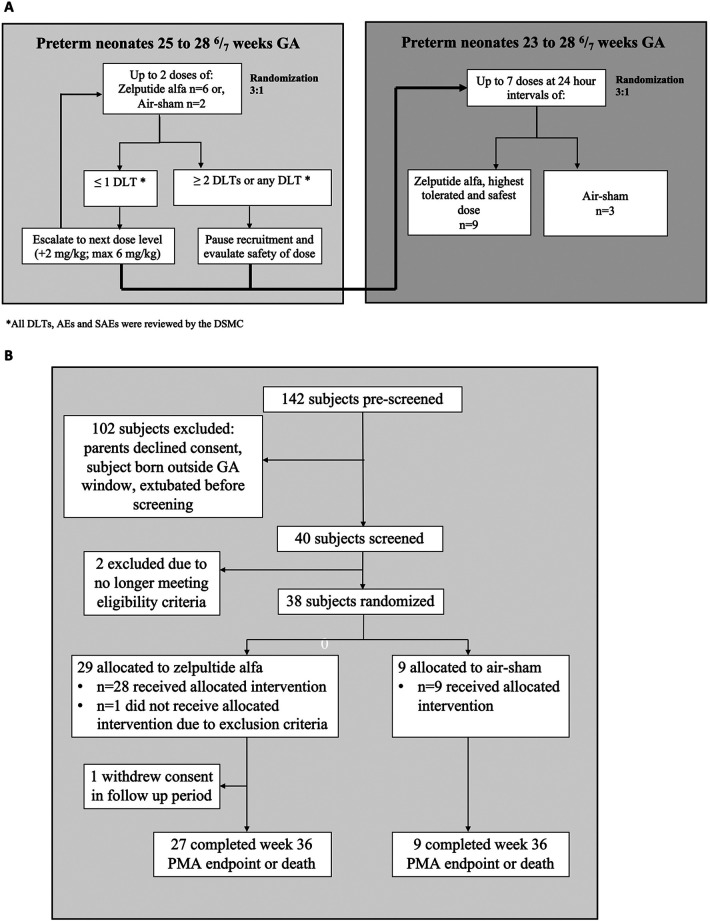
**(A)** Study diagram showing the two study phases. Initially, eight subjects (25–28 ^6^/_7_ weeks GA) were randomized 3:1 to receive up to two doses of intratracheal zelpultide alfa at each dosing level (2, 4, or 6 mg/kg) or air-sham 24 h apart. Furthermore, 12 additional subjects (23–28 ^6^/_7_ weeks GA) were randomized 3:1 to receive the highest-tolerated dose of zelpultide alfa, or air-sham, once daily for up to 7 days. DLTs were the determining factor for whether new subjects could receive a higher dose of zelpultide alfa and whether additional subjects could subsequently be enrolled in the study. Continued dosing for an individual was paused if a DLT event occurred and was referred to the DSMC. **(B)** Patient flow diagram of the 38 randomized preterm neonates included in the intent-to-treat analysis.

This study had two phases. In the first phase, extremely preterm neonates (born between 25 and 28 ^6^/_7_ weeks GA) were randomized 3:1 in a sequential collective cohort approach following a classical dose-determining study design (12). Thus, six neonates received the standard of care (SOC) along with intratracheally administered zelpultide alfa at each dose level (2, 4, or 6 mg/kg, see Figure 1A), starting with 2 mg/kg, while two neonates received the SOC and an air-sham (1 mL of air drawn into a dosing syringe for intratracheal instillation). Randomized subjects received up to two doses of the study treatment, administered 24 h (±1 h) apart, provided they remained intubated for the second dose. The first dose of zelpultide alfa or air-sham was given through the endotracheal tube (ETT) at least 15 min after Curosurf® (Chiesi Farmaceutici Spa, Parma, Italy) surfactant treatment and within 96 h of birth. In the second phase, once the highest-tolerated dose was confirmed, extremely preterm neonates (born between 23 and 28 ^6^/_7_ weeks GA, see Figure 1A) were enrolled concurrently and randomized in a 3:1 ratio, with nine receiving zelpultide alfa and three receiving the air-sham. Subjects could receive up to seven doses of zelpultide alfa or air-sham at approximately 24 h (±1 h) intervals, provided they remained intubated according to the SOC.

Zelpultide alfa was provided as a lyophilized powder for reconstitution at 4 mg/mL before intratracheal administration. The volume to be administered was determined by the subject's birth weight and dose level (2, 4, or 6 mg/kg). Subjects randomized to air-sham received 1 mL of room air regardless of their birth weight.

Dose-limiting toxicities (DLTs) determined whether new subjects could receive a higher dose of zelpultide alfa and whether additional subjects could subsequently be enrolled in the study. Continued dosing for a subject was paused if a DLT event occurred and was referred to the DSMC. All the subjects received the SOC, including, but not limited to, pulmonary surfactant, respiratory support, and nutrition, as per local hospital clinical guidelines. Study participation did not influence the SOC or lead to changes in the clinical management of the enrolled subjects. Follow-up occurred at day 28, week 36 postmenstrual age (PMA), hospital discharge, and months 6 and 12 of life for all subjects (the 6- and 12-month data are not reported in this publication). The healthcare team, investigators, subjects, and their parents were blinded to randomization. However, due to the nature of intratracheal treatment in the neonatal intensive care unit (NICU), the staff responsible for dose preparation and administration of zelpultide alfa and the air-sham were aware of the randomized assignments. These individuals conducted the dosing process out of sight and were requested not to disclose each subject's randomization to blinded staff.

### Study subjects

In the first phase of the trial (which included dose escalation and up to 2 days of treatment), 24 subjects born between 25 and 28 ^6^/_7_ weeks GA were planned to be enrolled, with eight subjects at each of the three dosing levels (six allocated to the zelpultide alfa group and two to the air-sham group). In the second phase of the trial (which tested up to 7 days of treatment), 12 subjects born between 23 and 28 ^6^/_7_ weeks GA were planned to be enrolled. Due to an error in the randomization system, one additional subject was randomized to the first phase 2 mg/kg zelpultide alfa-treatment group. After randomization but before treatment administration, one subject no longer met the eligibility criteria and was withdrawn from the study and replaced. Thus, 38 subjects were randomized, and 37 received treatment (Figure 1). The eligibility criteria included endotracheal intubation, mechanical ventilation, and receipt of at least one dose of surfactant treatment (Curosurf®) as part of the SOC after birth; the study population was limited to subjects who received Curosurf® to reduce variability related to surfactant choice. Subjects were excluded if birth weight was <400 or >1,800 g or if they had congenital abnormalities that impacted cardiovascular and pulmonary function. Subjects were also excluded if their birth mother was receiving chemotherapy or had hepatitis B, C, or E; HIV; a known active cytomegalovirus; COVID-19; a sexually transmitted infection; or a history of alcohol or drug abuse. The 37 randomized subjects treated were included in the intention-to-treat population.

### Ethics

The study was conducted in accordance with the Helsinki Declaration of 1975 (as amended in Edinburgh, Tokyo, Venice, Hong Kong, and South Africa), the International Council on Harmonization guidelines, or the laws and regulations of the country where the research took place, whichever provided greater protection to the study subjects. The ethics committee for the Spanish centers (Comité de Ética e Investigación Médica and Instituto de Investigación Sanitaria La Fe) and WCG IRB (tracking number 20210738) for the US centers reviewed and approved the protocol of the study. Approval was also secured from local and national medical research council ethics committees, along with the regulatory authorities. The parents or guardians of the subjects provided written informed consent prior to any study-related procedures. For the studies conducted under the United States’ Investigational New Drug law, the fundamental principles of Good Clinical Practice were followed, as outlined in 21 CFR 312, subpart D, “Responsibilities of Sponsors and Investigators,” 21 CFR, Part 50, 1998, and 21 CFR, Part 56, 1998. This study was conducted in compliance with 21 CFR, Part 320, 1993, “Retention of Bioavailability and Bioequivalence Testing Samples.”

### Outcomes

The primary outcome was the safety and tolerability of zelpultide alfa. Safety outcomes included the incidence of adverse events (AEs), serious adverse events (SAEs), and hematology and serum chemistry lab abnormalities. Monitoring for safety included clinical measurements 15 min before treatment, at the time of treatment, and 15 min, 1 h, 2 h, 8 h, and 24 h post-treatment on the first 7 study days after the first dose; long-term follow-up included measurements at day 28 after birth and week 36 PMA. The safety parameters measured included vital signs, physical exam, FiO2, screening for cardiac arrhythmia, hematology, and serum chemistry labs, documenting concomitant medications and procedures, and ventilation status, including ventilation parameters [e.g., peak inspiratory pressure (PIP), positive end-expiratory pressure (PEEP), and tidal volume (TV)]. Immunogenicity was assessed at baseline, day 7, day 28 of life, and week 36 PMA. This information was registered in the electronic medical record. The AEs and SAEs are defined in the Supplementary Material. The DSMC evaluated the AEs, SAEs, DLTs, and the zelpultide alfa-related safety implications and recommended enrollment for escalation of dose levels or termination. The DLTs were distinguished from AEs if all of the following criteria were met: if the AE was possibly, probably, or directly related to zelpultide alfa administration; if the AE occurred at any time between the initial and last dose of zelpultide alfa +72 h; the AE was not directly related to surfactant administration or ETT placement error; the AE caused any of the following that were unstable after medical intervention or resulted in death: hemodynamic instability, shock or severe hypertension, partial pressure of carbon dioxide <25 or >65 mmHg, apnea, ETT/airway blockage that required immediate removal of ETT, hypoxia, extubation/reintubation resulting in death, or anaphylaxis.

The secondary outcomes were the preliminary efficacy outcomes and complications of prematurity. The key efficacy outcomes included the incidence of BPD or death at week 36 PMA and the time (days) of mechanical ventilation from birth to 36 weeks PMA among survivors. The definition and classification of BPD were based on Jensen et al. (13). Days of mechanical ventilation were defined as ≥12 h within a calendar day. Other secondary outcomes included postnatal steroids, number of days in hospital, immunogenicity, pharmacokinetic analysis, pneumonia, blood infection, incidence of retinopathy of prematurity (ROP), pulmonary hypertension, patent ductus arteriosus (PDA), necrotizing enterocolitis (NEC), or severe intraventricular hemorrhage (IVH). Secondary outcomes were analyzed for all the randomized subjects, assuming the best-case outcome for non-survivors; no post-analysis corrections were conducted since there was no statistical analysis, as indicated below. Exploratory endpoints included changes in inflammatory mediators potentially associated with lung injury and BPD through 36 weeks.

### Statistics

Since this was a phase 1b study, the sample size was not statistically powered for safety and efficacy-related outcomes. Thus, no inferential statistics were planned, and analyses of safety and efficacy endpoints were descriptive only. Continuous variables are presented as mean, standard deviation (SD), median, minimum, and maximum. The categorical variables are reported as the number and percentage of subjects. The incidence of the events for the efficacy endpoints was numerically compared between the zelpultide alfa and air-sham groups. Baseline results were defined as the closest measurements taken before the first dose of study medication.

## Results

The study was initiated in March 2022 and completed in August 2023 for the primary endpoint of safety and tolerability of zelpultide alfa; the study, including the long-term follow-up endpoints (up to 12 months of life), was completed in May 2024. A total of 142 subjects were pre-screened as potential participants, and 40 were screened. The reasons for preventing screening were declining consent, extubation shortly after birth, and not fulfilling GA requirements (Figure 1B). Of the preterm neonates who received treatment (in the first phase, zelpultide alfa 2 mg/kg (*n* = 7), zelpultide alfa 4 mg/kg (*n* = 6), zelpultide alfa 6 mg/kg (*n* = 6), and air-sham (*n* = 6); in the second phase, zelpultide alfa 6 mg/kg (*n* = 9), and air-sham (*n* = 3), 36 completed the week 36 PMA or death endpoint. One subject discontinued the study before week 36 PMA due to withdrawal of consent (in the first phase, zelpultide alfa 2 mg/kg, *n* = 1). The general characteristics of the population and ventilatory support are shown in Table 1. No significant differences for these parameters were found.

**Table 1:** Baseline demographics, clinical characteristics, and ventilatory support in the first and second phases of the trial, and total.

Characteristics	First phase of trial			Second phase of trial	Total	
	Zelpultide alfa, 2 mg/kg (*n* = 7)	Zelpultide alfa, 4 mg/kg (*n* = 6)	Zelpultide alfa, 6 mg/kg (*n* = 6)	Zelpultide alfa, 6 mg/kg (*n* = 9)	All zelpultide alfa (*n* = 28)	Air-sham (*n* = 9)
GA (weeks)						
Median	26.0	26.0	27.8	25.9	26.1	25.6
Min, max	26, 27	26, 28	26, 29	23, 29	23, 29	24, 28
*N* (%)						
23	0 (0)	0 (0)	0 (0)	2 (22.2)	2 (7.1)	2 (22.2)
24	0 (0)	0 (0)	0 (0)	2 (22.2)	2 (7.1)	0 (0)
25	3 (43)	3 (50)	1 (16.7)	1 (11.1)	8 (28.6)	4 (44.4)
26	4 (57)	2 (33)	1 (16.7)	2 (22.2)	9 (32.1)	0 (0)
27	0 (0)	0 (0)	1 (16.7)	1 (11.1)	2 (7.1)	2 (22.2)
28	0 (0)	1 (17)	3 (50)	1 (11.1)	5 (17.9)	1 (11.1)
Birthweight, *g*, mean (SD)	793 (177)	804 (229)	990 (307)	696 (268)	806 (259)	741 (247)
Sex, male, *n* (%)	4 (57.1)	4 (66.7)	4 (66.7)	7 (77.8)	19 (67.9)	6 (66.7)
Race, *n* (%)						
Caucasian	5 (71.4)	5 (83.3)	4 (66.7)	6 (66.7)	20 (71.4)	5 (55.6)
Black or African American	1 (14.3)	0	2 (33.3)	2 (22.2)	5 (17.9)	1 (11.1)
Asian	0	0	0	0	1 (3.6)	0
Other	1 (14.3)	1 (16.7)	0	0	2 (7.1)	2 (22.2)
Congenital anomalies,^a^ *n* (%)	3 (42.9)	0	4 (66.7)	2 (22.2)	9 (32.1)	1 (11.1)
Maternal antenatal steroids, *n* (%)	2 (28.6)	4 (66.7)	2 (33.3)	4 (44.4)	12 (42.9)	3 (33.3)
Baseline respiratory support						
Ventilatory mode, *n* (%)						
AC	0 (0)	0 (0)	0 (0)	0 (0)	0 (0)	0 (0)
HFV	2 (28.6)	3 (50)	0 (0)	3 (33.3)	8 (28.6)	2 (22.2)
IMV	0 (0)	0 (0)	0 (0)	0 (0)	0 (0)	1 (11.1)
PCV	2 (28.6)	3 (50)	5 (83.3)	4 (44.4)	14 (50)	4 (44.4)
VCV	3 (42.9)	0 (0)	1 (16.7)	2 (22.2)	6 (21.4)	2 (22.2)
MAP, cmH2O, mean (SD)	9.7 (2.61)	10.1 (2.83)	7.4 (1.73)	8.1 (1.91)	8.8 (2.42)	9.1 (1.67)
FiO2, %, mean (SD)	21.9 (1.46)	31.0 (12.66)	26.8 (11.46)	23.3 (3.16)	25.4 (8.34)	28.8 (9.73)

AC, assist-control mode; HFV, high frequency ventilation (jet or oscillatory); FiO2, fraction of inspired oxygen; GA, gestational age; IMV, intermittent mechanical ventilation; MAP, mean airway pressure; PCV, pressure-targeted conventional ventilation; SD, standard deviation; VCV, volume-targeted conventional ventilation.

The reporting format of the minimum and maximum intervals for the GA was rounded to 1 significant digit without decimals.

^a^ The congenital disorders measured were as follows: hypospadias, adrenal insufficiency neonatal, adrenogenital syndrome, congenital ankyloglossia, atrial septal defect, congenital cytomegalovirus infection, exomphalos, homocystinuria, microcephaly, sickle cell trait, and talipes.

### Safety outcomes

Dose escalation proceeded to the highest dose, with no DLTs reported. Thus, the dose administered in the second phase was 6 mg/kg of zelpultide alfa. As shown in Table 2, 92.9% of all subjects treated with zelpultide alfa experienced at least one AE compared with 100% of those treated with air-sham. The most frequently reported AEs were anemia, PDA, ROP, hyperbilirubinemia, hyperglycemia, and hypotension. Table 3 describes the AEs reported in >10% of subjects in any treatment arm from the first treatment to week 36 PMA. Four subjects in the zelpultide alfa arms of the first phase (2 mg/kg, *n* = 2; 4 mg/kg, *n* = 2) and three subjects in the second phase of the trial (6 mg/kg up to seven doses, *n* = 3) experienced a total of nine AEs that were considered related to the medication. Eight of these AEs were considered possibly related to treatment, and one was considered hypoxia caused by the procedure. No AEs related to the treatment in the zelpultide alfa 6 mg/kg group in the first phase of the trial or the air-sham treatment groups were reported. In total, 17 subjects experienced 26 SAEs (Supplementary Table S1). Only two SAEs (pulmonary hemorrhage) were assessed as possibly related to treatment by the investigator; however, the study medical monitor and the Sponsor’s medical monitor related them to extreme prematurity-associated complications. The other 24 SAEs were unrelated to the study drug. Four subjects treated with zelpultide alfa experienced SAEs that led to withdrawal of the study drug (pulmonary hemorrhage, respiratory failure, sepsis, and IVH); three out of these were considered unrelated to treatment.

**Table 2:** Summary of AEs and SAEs.

*n* (%)	First phase of trial			Second phase of trial	Total	
	Zelpultide alfa, 2 mg/kg (*n* = 7)	Zelpultide alfa, 4 mg/kg (*n* = 6)	Zelpultide alfa, 6 mg/kg (*n* = 6)	Zelpultide alfa, 6 mg/kg (*n* = 9)	All zelpultide alfa (*n* = 28)	Air-sham (*n* = 9)
At least one AE	7 (100.0)	4 (66.7)	6 (100.0)	9 (100.0)	26 (92.9)	9 (100.0)
At least one SAE	4 (57.1)	2 (33.3)	1 (16.7)	6 (66.7)	13 (46.4)	4 (44.4)
At least one AE leading to discontinuation of the study drug	1 (14.3)	0	1 (16.7)	2 (22.2)	4 (14.3)	0

AE, adverse event; SAE, serious adverse event.

The number of patients experiencing AEs and SAEs in each treatment arm from the first treatment to week 36 PMA is presented with percentages in brackets.

**Table 3:** AEs reported in more than 10% of subjects in any treatment arm from first treatment to week 36 PMA.

AE reported	Trial first phase			Trial second phase	Total		*p*
	Zelpultide alfa, 2 mg/kg (*n* = 7)	Zelpultide alfa, 4 mg/kg (*n* = 6)	Zelpultide alfa, 6 mg/kg (*n* = 6)	Zelpultide alfa, 6 mg/kg (*n* = 9)	Zelpultide alfa (*n* = 28)	Air-sham (*n* = 9)	
Possibly related to the intervention							
Hypoxia/oxygen saturation decreased (%)	0	0	0	2 (22.2)	2 (7.1)	0	0.41
Pulmonary hemorrhage (%)	1 (14.3)	1 (16.7)	0	1 (11.1)	3 (10.7)	0	0.31
Prolonged QT interval (%)	0	1 (16.7)	0	0	1 (3.6)	0	0.57
Bradycardia (HR < 100 bpm) (%)	0	0	0	1 (11.1)	1 (3.6)	0	0.57
Not related to the intervention							
Hypoxia/oxygen saturation decreased (%)	2 (28.6)	1 (16.7)	0	0	3 (10.7)	0	0.31
Pulmonary hemorrhage (%)	0	0	0	0	0	1 (11.1)	0.07
Pulmonary interstitial emphysema (%)	1 (14.3)	0	0	2 (22.2)	3 (10.7)	1 (11.1)	0.97
Respiratory failure (%)	1 (14.3)	0	1 (16.7)	0	2 (7.1)	1 (11.1)	0.71
Pulmonary hypertension (%)	0	0	1 (16.7)	0	1 (3.6)	1 (11.1)	0.38
Pulmonary edema (%)	1 (14.3)^a^	0	3 (50.0)^a^	3 (33.3)^a^	7 (25.0)^a^	0	0.10
BPD (%)	0	1 (16.7)	1 (16.7)	0	2 (7.1)	1 (11.1)	0.70
Anemia (%)	4 (57.2)	2 (33.3)	2 (33.3)	4 (44.4)	12 (42.8)	6 (66.6)	0.21
Thrombocytopenia (%)	1 (14.3)	0	0	1 (11.1)	2 (7.1)	1 (11.1)	0.71
Hyperglycemia (%)	1 (14.3)	0	2 (33.3)	4 (44.4)	7 (25.0)	1 (11.1)	0.38
Electrolyte imbalance (%)	1 (14.3)	2 (33.3)	2 (33.3)	4 (44.4)	9 (32.2)	2 (22.2)	0.57
Metabolic acidosis (%)	1 (14.3)	0	0	2 (22.2)	3 (10.7)	0	0.31
Severe nosocomial infection (%)	1 (14.3)	0	0	1 (11.1)	2 (7.1)	5 (55.5)	0.001
Conjunctivitis (%)	1 (14.3)	0	0	0	1 (3.6)	1 (11.1)	0.38
Bronchiolitis (%)	0	0	0	0	0	1 (11.1)	0.07
Eye infection (%)	0	0	0	0	0	1 (11.1)	0.07
Gastrointestinal disorders (%)	4 (57.1)	2 (33.3)	0	5 (55.6)	11 (39.3)	4 (44.4)	0.78
Inguinal hernia (%)	1 (14.3)	1 (16.7)	0	1 (11.1)	3 (10.7)	1 (11.1)	0.97
Intestinal perforation (%)	1 (14.3)	0	0	2 (22.2)	3 (10.7)	1 (11.1)	0.97
Abdominal distension (%)	1 (14.3)	0	0	2 (22.2)	3 (10.7)	0	0.31
Neonatal intestinal perforation (%)	1 (14.3)	0	0	1 (11.1)	2 (7.1)	1 (11.1)	0.71
NEC (%)	1 (14.3)	0	0	0	1 (3.6)	1 (11.1)	0.38
PDA (%)	3 (42.9)	2 (33.3)	2 (33.3)	3 (33.3)	10 (35.7)	3 (33.3)	0.90
ROP (%)	3 (42.9)	0	2 (33.3)	2 (22.2)	7 (25.0)	3 (33.3)	0.62
Hyperbilirubinemia (%)	2 (28.6)	1 (16.7)	3 (50.0)	2 (22.2)	8 (28.6)	1 (11.1)	0.29
IVH grade 2 (%)	2 (28.6)	2 (33.3)	0	2 (22.2)	6 (21.4)	1 (11.1)	0.49
Renal failure (%)	1 (14.3)	0	1 (16.7)	3 (33.3)	5 (17.9)	1 (11.1)	0.63
Adrenal insufficiency (%)	0	0	2 (33.3)	3 (33.3)	5 (17.9)	0	0.17

AE, adverse event; BPD, bronchopulmonary dysplasia; IVH, intraventricular hemorrhage; NEC, necrotizing enterocolitis; PDA, patent ductus arteriosus; PMA, postmenstrual age; ROP, retinopathy of prematurity.

The number of patients experiencing AEs in each treatment arm is presented with percentages in brackets. Severe nosocomial infection included infections with a positive culture from blood or cerebrospinal fluid.

^a^ Further investigation of these events concluded that pulmonary edemas should not have been recorded as AEs, as all events happened at the same site where furosemide was given as the standard of care. These should have been reported as concomitant medication instead of as an AE. Therefore, the incidence of pulmonary edema has been corrected to 0 in all cases where pulmonary edema was reported.

Supplementary Table S2 describes the characteristics of the six subjects who died and the complications or interventions that led to death in the intervention group. Supplementary Table S2 also includes the date of the last dose of zelpultide alfa, the date of diagnosis of the complication or intervention, and the time elapsed between both. No differences in baseline characteristics were found between infants who died and those who survived.

Supplementary Table S3 presents the pharmacokinetic analysis. No patient developed anti-SP-D antibodies (anti-zelpultide alfa) after being treated with zelpultide alfa through week 36 PMA. Dose-dependent increases in blood SP-D concentrations were observed in subjects treated with zelpultide alfa in the 24 h after the first treatment dose compared to baseline, and these were highest with the administration of the 6 mg/kg dose (Supplementary Table S3).

### Efficacy outcomes

**Figure 2:**
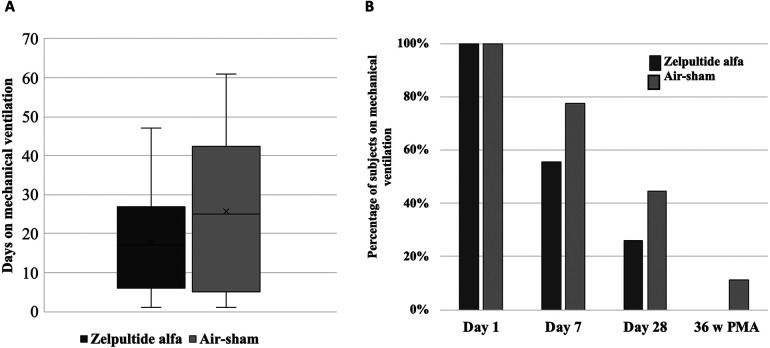
**(A)** Time (days) on mechanical ventilation of the subjects treated with zelpultide alfa vs. air-sham at week 36 PMA among survivors. In total, 30 patients reached week 36 PMA (21 treated with zelpultide alfa, 9 treated with air-sham). **(B)** Percentage of survivors who were receiving mechanical ventilation at each study time point. For zelpultide alfa, at days 1, 7, and 28 and week 36 PMA, there were 28, 26, 23, and 22 survivors, respectively. For air-sham, there were nine survivors at all visits. The subjects who died were excluded.

Supplementary Table S4 shows that preterm infants in the zelpultide alfa group had 32.1% vs. 66.7% incidence of BPD, 53.6% vs. 66.7% incidence of BPD or death, 39% vs. 56% incidence of grade 2 or 3 BPD or death, and no cases vs. 11.1% of grade 3 BPD compared to the air-sham group. As shown in Figure 2A, patients in the zelpultide alfa group were on mechanical ventilation for 17.7 days compared with 25.8 days in the air-sham group. Moreover, as shown in Figure 2B, no patients who survived in the zelpultide alfa group required mechanical ventilation at 36 weeks PMA, while 11% in the air-sham group were still ventilated at this time point. The need for mechanical ventilation was less frequent in the zelpultide alfa arm than in the air-sham group at each time point measured between birth and 36 weeks GA.

### Additional outcomes

Results for additional outcomes are reflected in Supplementary Table S5.

## Discussion

In this clinical trial, the highest tested dose of intratracheal zelpultide alfa (6 mg/kg administered for up to 7 days) was considered safe and well-tolerated when added to the SOC for the treatment of extremely preterm neonates at high risk of developing BPD. No DLTs were reported in the subjects who received zelpultide alfa. The DSMC assessed the 6 mg/kg dose to be safe and recommended it for progression in future studies. Due to statistical limitations and potential attrition bias, efficacy conclusions regarding the impact of zelpultide alfa on respiratory outcomes were not possible.

Six patients in the zelpultide alfa group (21.4%) and no patients (0%) in the air-sham group died while in the NICU. As shown in Supplementary Table S2, death was caused by bacterial sepsis (7.1%), intestinal perforation during an intervention for congenital omphalocele, medication for PDA closure, stent insertion (10.7%), and bilateral IVH (3.6%). The mortality rate in the intervention group was 21.4%, and the mortality-related conditions are within the scope described for extremely preterm infants in high-income countries (HICs) (14). The time elapsed between the last dose of zelpultide alfa and the conditions/interventions that triggered the patients' deaths is relevant for establishing an association or a causative effect. As shown in Supplementary Table S2, the time elapsed between the intervention and the diagnosis of the condition that led to death was >3 days in four out of the six cases that died. Intriguingly, no patient in the air-sham group with similar perinatal characteristics to the intervention group (Table 1) died, whereas the mortality in this population, according to published reports, should have ranged between 11% and 30% in HICs (14–18). We do not have a satisfactory answer for the lack of mortality in the air-sham group; however, a feasible explanation for these results could be attributed to the ethical considerations of the study design, which required a reduced number of patients in the air-sham group.

The secondary efficacy outcomes were designed to determine whether zelpultide alfa therapy could influence the combined outcomes of BPD and death and/or time on respiratory support through 36 weeks PMA. The determination of these endpoints and obtaining preliminary data were relevant for applying them in future efficacy clinical studies. Grades 2 and 3 BPD (moderate to severe) were chosen as an outcome, as babies in these categories have the highest risk of extensive morbidities and worse long-term outcomes and place a significant burden on intensive care services (18, 19). An additional analysis was performed excluding deaths before 14 days of life, which is a meaningful analysis due to the high rate of mortality within this period as a consequence of the comorbidities associated with prematurity (20). In recent years, there has been a focus on avoiding unnecessary invasive ventilation in preterm neonates to prevent long-term lung injury (19). Although ventilation strategies have improved, mechanical ventilation increases lung tissue injury, blunts secondary alveolar septation (21), increases the risk of ventilator-associated pneumonia (22), activates pro-inflammatory cascades, and increases the risk of BPD (1). Thus, reducing the time spent on mechanical ventilation is relevant for preventing BPD (18).

There were several limitations in this trial related to its design. A blinded clinical trial is the gold standard for limiting bias in study design. However, blinding to treatment in a NICU is challenging when using an intratracheally administered product within a limited timeframe. The use of a placebo also limits bias in any study design. However, a placebo administered intratracheally in the NICU setting is not ethical or practical. Therefore, the SOC with air-sham was chosen as the comparator in this trial. Although air-sham as a comparator is not completely without risks associated with intratracheal use, the risks to the subjects were considered minimal. Thus, the individuals who prepared and administered the dose were aware of the treatment allocation; however, all other NICU healthcare staff, investigators, subjects, and their parents were intended to remain blinded to treatment. In the last decade, there has been a tendency toward early extubation and earlier transition to non-invasive ventilation (10). The study protocol determined the maximum number of doses a subject could receive; however, the actual number was determined by clinical indication for continuation of intubation, and therefore, the number of doses received varied among patients. Moreover, tracheal intubation for mechanical ventilation was mandatory. Thus, there was variability in the number of treatment administrations received by each subject, depending on when the subjects were extubated, as per the SOC. In the second phase, where the treatment could be administered for up to 7 days, over 66% of subjects received ≥5 administrations. This limitation was difficult to overcome because the study aimed not to interfere with the SOC in the respiratory management of the subjects. In addition, the 3:1 randomization chosen for ethical considerations made the comparator group (air-sham) very small and could have introduced bias in the mortality rate.

## Conclusions

The results from this phase 1b study of intratracheally administered zelpultide alfa in extremely preterm neonates at a dose of 6 mg/kg for up to 7 days showed that this therapy is safe and well-tolerated. Although the mortality rate was higher in the treatment group, it was within the normal range for extremely preterm infants in high-income countries. Consequently, this was the DSMC-recommended dose for future studies. Although the study was not powered for efficacy, the incidence of BPD or death was similar in the air-sham and zelpultide alfa groups. However, other respiratory-related outcomes, such as grade 2 or 3 BPD or death, were reduced by 17%, and time on mechanical ventilation after 36 weeks PMA was reduced by 31% in the zelpultide alfa group compared to the air-sham group, despite the difference in mortality in the former. These results warrant a larger and adequately powered randomized controlled trial to assess the effectiveness of zelpultide alfa in preventing BPD in extremely preterm infants.

## Data Availability

The original contributions presented in the study are included in the article/Supplementary Material, further inquiries can be directed to the corresponding author.
