# Supplementary material for: A phase 1b randomized, multicenter, dose determination trial of zelpultide alfa (recombinant human surfactant protein D) in preterm neonates at high risk of developing bronchopulmonary dysplasia
Source: Front Pediatr. 2025 Sep 12;13:1639573. doi: 10.3389/fped.2025.1639573 (PMC12465625; doi:10.3389/fped.2025.1639573)
Supplement: Supplementary file 1 [file Supplementaryfile1.docx]

# **Supplemental Information**

List of study sites and investigators for each center that collaborated in the recruitment of patients: Manoj Biniwale, PIH Health Good Samaritan Hospital (U.S.A.); Jamil Khan, Children’s Hospital of the King’s Daughters (U.S.A.); María Luz Couce-Pico, Complejo Hospitalario Universitario de Santiago (Spain); Ana María Sánchez-Torres, Hospital Universitario La Paz (Spain); Caridad Tapia-Collados, Hospital General Universitario Alicante Doctor Balmis (Spain); Sherry Courtney, Arkansas Children’s Hospital/ The University of Arkansas for Medical Sciences (U.S.A.)

Additional contributors and collaborators to the study: Cristina García-Mouton, Shawn Grant, Paul Kingma, Neil P. Mills, Petra Roulhac, Tal Scheuer, Alan Wolk

**Adverse Event (AE) and Serious Adverse Event (SAE) definitions:**

According to the protocol of the study, the definitions used for AEs and SAEs were:

“An adverse event (AE) is any untoward, undesired, or unplanned event in the form of signs, symptoms, disease, or laboratory or physiologic observations occurring in a person administered an investigational product in a clinical study. The event does not need to be causally related to an investigational product. An AE must only be classified as serious, i.e., an SAE, when the event meets one of the following criteria: i) Results in death, ii) Is life-threatening (refers to an event in which the subject was at risk of death at the

time of the event; it does not refer to an event which, hypothetically, might have

caused death if it were more severe), iii) Requires inpatient hospitalization or prolongation of existing hospitalization, iv) Results in persistent or significant disability/incapacity, v) Is a congenital anomaly/birth defect, vi) Is medically significant (these events may not be immediately life-threatening or result in death or hospitalization but may jeopardize the patient or may require intervention to prevent one of the other outcomes listed in the definition above.

The relationship of each AE to the investigational product was recorded as Unrelated (Not Related), Possibly Related, Probably Related, or Directly Related using the following definitions:

- *Unrelated (Not related):* The AE is clearly not related to the investigational agent(s) or intervention. The AE has no temporal relationship to the administration of the investigational agent(s) or research intervention and follows no known or suspected pattern of response, and an alternative cause is present.
- *Possibly Related:* The AE is possibly related to the investigational agent(s) or research intervention. The AE may follow no known pattern of response, and an alternative cause seems more likely.
- *Probably Related:* The AE is probably related to the investigational agent(s) or research intervention. The AE has a timely relationship to the administration of the investigational agent(s) or research intervention and follows a known pattern of response, but a potential alternative cause may be present.
- *Directly Related:* The AE is clearly related to the investigational agent(s) or research intervention. The AE has a temporal relationship to the administration of the investigational agent(s) or research intervention and follows a known pattern of response, and no alternative cause is present.

**Table S1.** SAEs reported from the first treatment to week 36 PMA in any treatment arm. The number of patients experiencing SAEs in each treatment arm is presented with percentages in brackets.

|  | Trial first phase | | | Trial second phase | Total | |
| --- | --- | --- | --- | --- | --- | --- |
| n (%) | zelpultide alfa, 2 mg/kg  (n=7) | zelpultide alfa,  4 mg/kg  (n=6) | zelpultide alfa,  6 mg/kg  (n=6) | zelpultide alfa,  6 mg/kg  (n=9) | All zelpultide alfa  (n=28) | Air-sham  (n=9) |
| Intestinal perforation | 1 (14.3) | 0 | 0 | 2 (22.2) * | 3 (10.7) | 0 |
| Neonatal intestinal perforation | 1 (14.3) | 0 | 0 | 1 (11.1) | 2 (7.1) | 1 (11.1) |
| Pulmonary hemorrhage | 1 (14.3) | 0 | 0 | 1 (11.1) | 2 (7.1) | 0 |
| IVH | 0 | 0 | 0 | 2 (22.2) * | 2 (7.1) | 0 |
| NEC | 0 | 0 | 0 | 0 | 0 | 1 (11.1) |
| Small intestinal obstruction | 1 (14.3) | 0 | 0 | 0 | 1 (3.6) | 0 |
| Bronchiolitis | 0 | 0 | 0 | 0 | 0 | 1 (11.1) |
| Enterobacter sepsis | 0 | 0 | 0 | 1 (11.1) * | 1 (3.6) | 0 |
| Meningitis staphylococcal | 0 | 0 | 0 | 0 | 0 | 1 (11.1) |
| Pneumonia bacterial | 0 | 0 | 0 | 0 | 0 | 1 (11.1) |
| Pneumonia staphylococcal | 0 | 0 | 0 | 0 | 0 | 1 (11.1) |
| Sepsis | 0 | 0 | 0 | 1 (11.1) * | 1 (3.6) | 0 |
| Septic shock | 0 | 0 | 0 | 0 | 0 | 1 (11.1) |
| Pulmonary arterial hypertension | 0 | 1 (16.7) | 0 | 0 | 1 (3.6) | 0 |
| Respiratory failure | 0 | 0 | 1 (16.7) * | 0 | 1 (3.6) | 0 |
| Status epilepticus | 0 | 1 (16.7) | 0 | 0 | 1 (3.6) | 0 |
| Ventricular tachycardia | 0 | 0 | 0 | 1 (11.1) | 1 (3.6) | 0 |
| PDA | 1 (14.3) * | 0 | 0 | 0 | 1 (3.6) | 0 |
| Hyperkalemia | 0 | 0 | 0 | 1 (11.1) | 1 (3.6) | 0 |
| Lactic acidosis | 0 | 0 | 0 | 1 (11.1) | 1 (3.6) | 0 |

Abbreviations: IVH, intraventricular hemorrhage; NEC, necrotizing enterocolitis; PDA, patent ductus arteriosus; PMA, postmenstrual age; SAE, serious adverse event. *Indicates SAEs that led to death; none of the six cases were related to the study drug.

**Table S2.** Descriptive analysis of preterm infants who died during the trial, conditions/interventions that caused death, and time elapsed between the administration of Zelpultide alfa and onset of the condition/intervention that led to death.

| **Case** | **Gestational age**  **(weeks)** | **Birth weight** | **Sex** | **Race** | **Cohort** | **Interventionor conditions**  **that led to death** | **Date of**  **last dose of**  **zelpultide alfa** | **Onset date of condition or intervention leading to death** | **Time**  **elapsed between last dose zelpultide alfa**  **and onset of**  **death cause**  **(days)** |
| --- | --- | --- | --- | --- | --- | --- | --- | --- | --- |
| Case 1 | 25w 6/7d | 820g | M | White | Zel2 | Stent-induced aortic obstruction during intervention for PDA | 10-Sep-22 | 30-Sep-22 | 20 |
| Case 2 | 25w 4/7d | 900g | M | White | Zel6 | Respiratory Failure | 19-Jan-23 | 23-Jan-23 | 4 |
| Case 3 | 23w 0/7d | 510g | F | Black or  AA | Zel2nd | Intestinal perforation secondary to PDA closure treatment | 20-Apr-23 | 28-Apr-23 | 8 |
| Case 4 | 23w 2/7d | 515g | M | White | Zel2nd | LOS | 19-Apr-23 | 1-May-23 | 12 |
| Case 5 | 24w 4/7d | 850g | M | Black or  AA | Zel2nd | Bilateral IVH  grade IV | 24-May-23 | 25-May-23 | 1 |
| Case 6 | 25w 6/7d | 500g | M | White | Zel2nd | LOS | 23-May-23 | 24-May-23 | 1 |

Abbreviations: AA: African-American; F: female; GA: gestational age; LOS: late-onset sepsis; IVH: intraventricular hemorrhage; M: male; PDA: persistent ductus arteriosus; Zel2: treatment group with zelpultide alfa, 2 mg/kg; Zel4: treatment group with zelpultide alfa, 4 mg/kg; Zel2nd: treatment group second phase of the trial, zelpultide alfa 6 mg/kg.

**Table S3.** Pharmacokinetics of zelpultide alfa (ng/mL) and air-sham showing change in SP-D blood levels from baseline at day 1 for each treatment arm.

| n (%) | First phase of trial | | | Second phase of trial | Total |
| --- | --- | --- | --- | --- | --- |
|  | zelpultide alfa, 2 mg/kg  (n=7) | zelpultide alfa,  4 mg/kg  (n=6) | zelpultide alfa, 6 mg/kg  (n=6) | zelpultide alfa, 6 mg/kg  (n=9) | Air-sham |
| n | 1 | 3 | 3 | 0 | 4 |
| Mean (SD) | -2.87 (-) | 150.14 (182.549) | 312.74 (249.638) | - | 13.96 (20.440) |
| Median | -2.87 | 78.63 | 380.70 | - | 10.03 |
| Range (min, max) | -2.9, -2.9 | 14.2, 357.6 | 36.2, 521.4 | - | -6.5, 42.2 |

Abbreviations: Max, maximum; min, minimum; SD, standard deviation.

**Table S4.** Key efficacy outcomes included the incidence of BPD or death at week 36 PMA in all zelpultide alfa and air-sham treatment groups. The definition and classification of BPD was based on Jensen et al. 2019.^17^ Incidence of BPD and individual BPD grades were reported considering all subjects randomized without applying corrections for non-survivors.

| n (%) | All Zelpultide alfa | Air-sham |
| --- | --- | --- |
| Subjects | 28 | 9 |
| BPD or death | 15 (54%) | 6 (67%) |
| Incidence of BPD | 9 (32%) | 6 (67%) |
| BPD grade 1 | 4 (14.3%) | 1 (11.1%) |
| BPD grade 2 | 5 (17.9%) | 4 (44.4%) |
| BPD grade 3 | 0 (0%) | 1 (11%) |
| Death | 6 (21.4%) | 0 (0.0%) |
| BPD grade 2 or 3, or death | 11 (39%) | 5 (56%) |

Abbreviations: BPD, bronchopulmonary dysplasia.

**Table S5.** Additional outcomes and comorbidities that occurred in any treatment arm from first treatment to week 36 PMA. The number of patients experiencing outcomes and comorbidities in each treatment arm are presented with percentages in bracket, except days in hospital that are presented as mean (SD). Outcomes were reported considering all subjects randomized without applying corrections for non-survivors. For the mean number of days in hospital, there was a recalculation for number of days in hospital excluding subjects that died. Data from the 6 subjects that died by week 36 PMA was excluded from the analysis and resulted in 87.2 days (±24.1, n=21) for the all zelpultide alfa group and 90.2 days (±21.1, n=9) for the air-sham group.

|  | Trial first phase | | | Trial Second phase | Total | |
| --- | --- | --- | --- | --- | --- | --- |
| n (%) | zelpultide alfa,  2 mg/kg  (n=7) | zelpultide alfa  4 mg/kg  (n=6) | zelpultide alfa,  6 mg/kg  (n=6) | zelpultide alfa,  6 mg/kg  (n=9) | All zelpultide alfa  (n=28) | Air-sham  (n=9) |
| Postnatal steroids | 2 (28.6) | 2 (33.3) | 0 | 1 (11.1) | 5 (17.9) | 3 (33.3) |
| Hospital stays, days  mean (SD) | 76.3 (36.3) | 87.8 (8.9) | 81.5 (45.7) | 51.6 (39.1) | 72.7 (36.7) | 90.2 (21.1) |
| Pneumonia* | 0 | 0 | 0 | 0 | 0 | 1 (11.1) |
| Blood infection^†^ | 1 (14.3) | 3 (50.0) | 0 | 0 | 4 (14.3) | 2 (22.2) |
| Viral infection^‡^ | 0 | 1 (16.7) | 0 | 0 | 1 (3.6) | 0 |
| ROP | 3 (42.9) | 3 (50.0) | 2 (33.3) | 3 (33.3) | 11 (39.3) | 4 (44.4) |
| PDA | 3 (42.9) | 2 (33.3) | 2 (33.3) | 3 (33.3) | 10 (35.7) | 3 (33.3) |
| Pulmonary hypertension | 0 | 1 (16.7) | 1 (16.7) | 0 | 2 (7.1) | 1 (11.1) |
| NEC | 0 | 0 | 0 | 1 (11.1) | 1 (3.6) | 1 (11.1) |
| Grade 2 IVH | 0 | 0 | 1 (16.7) | 0 (0.0) | 1 (3.6) | 1 (11.1) |

Abbreviations: IVH, intraventricular hemorrhage; NEC, necrotizing enterocolitis; PDA, patent ductus arteriosus; ROP, retinopathy of prematurity; SD, standard deviation. *Confirmed by X-ray. †Confirmed by a positive blood culture. ‡Confirmed by culture or polymerase chain reaction test.
